# Identification of Immune Cell Infiltration and Immune-Related Genes in the Tumor Microenvironment of Glioblastomas

**DOI:** 10.3389/fimmu.2020.585034

**Published:** 2020-10-20

**Authors:** Sicong Huang, Zijun Song, Tiesong Zhang, Xuyan He, Kaiyuan Huang, Qihui Zhang, Jian Shen, Jianwei Pan

**Affiliations:** ^1^Department of Neurosurgery, The First Affiliated Hospital, Zhejiang University School of Medicine, Hangzhou, China; ^2^The First Affiliated Hospital, Institute of Translational Medicine, Zhejiang University School of Medicine, Hangzhou, China; ^3^The First Affiliated Hospital, School of Public Health, Zhejiang University School of Medicine, Hangzhou, China; ^4^Department of Neurology, Dong Fang Hospital, Beijing University of Chinese Medicine, Beijing, China; ^5^Department of Clinical Neurosciences, University of Calgary, Calgary, AB, Canada

**Keywords:** glioblastoma, tumor microenvironment, immune infiltration, immune therapy, TCGA

## Abstract

Glioblastoma (GBM) is one of the most prevalent malignant brain tumors with poor prognosis. Increasing evidence has revealed that infiltrating immune cells and other stromal components in the tumor microenvironment (TME) are associated with prognosis of GBM. The aim of the present study was to identify immune cells and immune-related genes extracted from TME in GBM. RNA-sequencing and clinical data of GBM were downloaded from The Cancer Genome Atlas (TCGA). Four survival-related immune cells were identified *via* Kaplan-Meier survival analysis and immune-related differentially expressed genes (DEGs) screened. Functional enrichment and protein-protein interaction (PPI) networks for the genes were constructed. In addition, we identified 24 hub genes and the expressions of 6 of the genes were significantly associated with prognosis of GBM. Finally, the genes were validated in single-cell sequencing studies of GBM, and the immune cells validated in an independent GBM cohort from the Chinese Glioma Genome Atlas (CGGA). Overall, 24 immune-related genes infiltrating the tumor microenvironment were identified in the present study, which could serve as novel biomarkers and immune therapeutic targets.

## Introduction

Glioblastoma (GBM) is the most common primary malignant brain tumor accounting for approximately 80% of all primary malignant brain tumors, and has a dismal prognosis and poor quality of life, with a median overall survival (OS) often < 1 year. Hereditary syndromes and ionizing radiation are the most common risk factors for GBM (1). The standard care of GBM is surgical resection followed by concomitant radiation therapy and chemotherapy with temozolomide (TMZ). Although multiple treatments have improved due to the development of gene therapy, immunotherapy, vaccine therapy, and others (2), therapeutic options for managing recurrence in GBM are limited. Immune checkpoint inhibitors (ICIs) such as anti-programmed cell death protein-1 (PD-1)/programmed death ligand-1 (PD-L1) and anti-cytotoxic T-lymphocyte-associated protein 4 (CTLA-4) have been extensively studied for both primary and recurrent glioblastomas in medical research. However, most of the clinical studies for GBM based on ICIs and trials with vaccine therapies have been unsuccessful. The cause of the failure in clinical trials of GBM *via* immunotherapy is attributed to several factors, including a highly immunosuppressive environment and multiple mechanisms of therapeutic resistance. GBM induces local immune dysfunction and systemic immunosuppression, which causes more complex coupling relationships between GBM and the surrounding tumor microenvironment (TME). Studying the mechanisms of GBM immunosuppression enhances our understanding on development of immunotherapy strategies (3).

TME is one of the crucial factors of local immune dysfunction, which establishes a niche for cancer cells, multiple stromal cells (endothelial cells, immune cells, etc.) and extracellular components (extracellular matrix, cytokines, growth factors, etc.). TME plays a critical role in the establishment of specific conditions, thereby interfering with angiogenesis, cell death, oxidative stress, and immune escape (4). Increasing studies have revealed that TME is not only pivotal in tumor initiation, progression, and migration, but it also affects generation of therapeutic resistance and malignancy. Cellular composition of TME and accessibility of immune cells exhibit large variations among GBM subtypes and patients. Such factors contribute to immunosuppression of GBM, which in turn lead to immunotherapeutic treatment failure (5). Identification of actively involved types of immune genes and immune cells associated with the TME facilitates elucidation of the general mechanisms of GBM immunosuppression.

Therefore, the present study investigated survival-related immune cells in GBM and identified hub genes associated with immune cell infiltration. We acquired RNA-sequencing (RNA-seq) expression data and corresponding clinical data of 166 patients with GBM from The Cancer Genome Atlas (TCGA) database. A total of 22 types of infiltrating immune cells in the 166 patients were estimated using the method of estimating relative subsets of RNA transcripts (CIBERSORT) (6). Subsequently, four survival-related immune cells were identified from the survival analyses of 22 types of immune cells. Immune-related genes were ranked through differential gene expression analyses and 24 hub genes selected from the protein-protein interaction (PPI) network established using Cytoscape (7). Six hub genes associated with overall survival were identified. Finally, immune cells were validated in an independent GBM cohort from the Chinese Glioma Genome Atlas (CGGA), and hub genes verified in single-cell sequencing studies of GBM. All analyses were conducted using R software. The findings of the present study provide valuable information that will guide patient-specific clinical immunotherapeutic strategies, and further construction of prediction models for prognosis of GBM. Moreover, immune cells infiltrating in the tumor microenvironment could act as therapeutic targets for the clinical treatment of GBM.

## Materials and Methods

### Raw Data Collection

**Figure 1:**
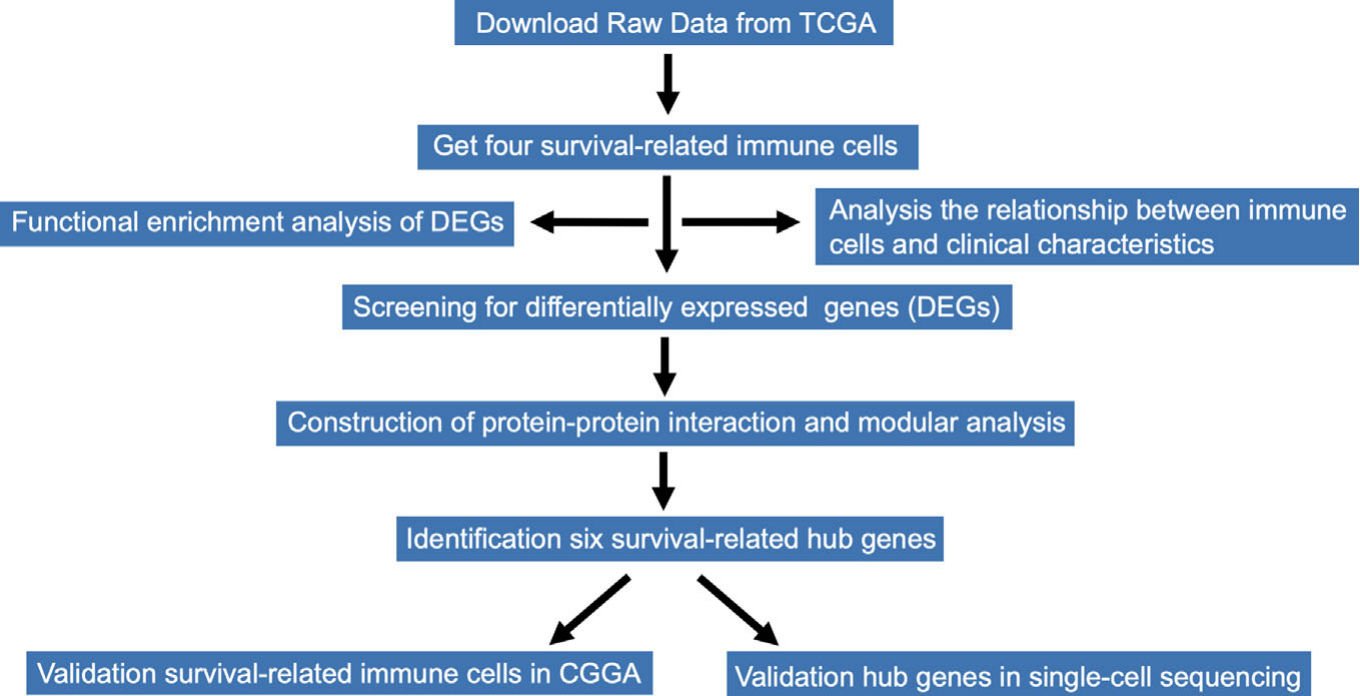
Flow chart of the whole analysis process.

RNA-Seq expression profiles of immune cells and corresponding clinical data of 166 patients with GBM were downloaded from TCGA database. The file format of RNA-seq expression was FPKM. The expression profile of each sample included age, gender, expression subclass, and MGMT promoter status. RNA-Seq expression information of immune cells from CGGA were also downloaded for the validation. Data acquisition and analyses were performed using R software (8).The entire research data analysis process is presented in **Figure 1**.

### Identification of Survival-Related Tumor-Infiltrating Immune Cells

CIBERSORT is an analytical algorithm, which can characterize cell composition of complex tissues based on normalized gene expression profiles (9). We used CIBERSORT to estimate the ratio of 22 infiltrating immune cell types based on each GBM sample. Afterward, 57 samples with P ≤ 0.05 were selected and correlation analyses conducted to analyze contents of the 22 immune cells (10). Survival analyses of the filtered immune cells in the tumor microenvironment were performed by the Kaplan-Meier survival analysis, with a cut-off level set at the median value. The results were tested by log-rank test. All the analyses were conducted using R software.

### Relationship Between Clinical Characteristics and Survival-Related Immune Cells

To determine the relationship between survival-related immune cells and clinical features such as age, gender, expression subclass, and MGMT promoter status, 57 samples were analyzed. An independent sample t-test was used to compare means of two groups, while one-way analysis of variance (ANOVA) test was used to compare the means of four groups.

### Identification and Functional Enrichment Analysis of Immune-Related Genes

Immune related-genes were analyzed using survival-related cells that had been obtained previously. Data analysis was performed using the edgeR R package, and |logFC| ≥ 1.0 and P < 0.05 were set as the cut-offs to screen for immune-related genes. Subsequently, a Venn diagram was used to visualize genes displayed by the online tool (http://bioinformatics.psb.ugent.be/webtools/Venn/) (11). DAVID software (https://david.ncifcrf.gov/) was used to analyze immune-related genes in the Gene Ontology(GO) terms and Kyoto Encyclopedia of Genes and Genomes (KEGG) pathways (12). Results of GO analysis revealed the functions of immune-related genes in biology process, cell component, and molecular function (13). KEGG pathway analyses results revealed the role of development-related signaling pathways.

### Construction of PPI Network, Selection and Analysis of Hub Genes

PPI networks of immune-related genes were predicted using the Search Tool for the Retrieval of Interacting Genes (STRING, https://string-db.org/) (14). An interaction combined score of >0.4 was considered statistically significant. Cytoscape is an open-access software platform designed to analyze and visualize complex interaction networks (7). Molecular Complex Detection(MCODE) plugin of Cytoscape was used to cluster the networks based on topology to identify densely connected regions with MCODE score > 5, degree cut-off = 2, node score cut-off = 0.2, max depth = 100, and k-score = 2 (15). Hub genes were defined based on module connectivity (16).

### Identification and Immune Infiltration of Survival-Related Hub Genes

Kaplan-Meier plots were used to identify immune-related genes in relation to the overall survival of patients. These results were analyzed by long-rank test. The correlation between 24 hub genes and 22 immune cells was determined using Person’s correlation analysis and CIBERSORT to reveal the relationship between hub genes and immune cells (17). Afterward, comprehensive correlation analysis between six selected survival-related hub genes and tumor-infiltrating immune cell signatures for GBM were performed using Tumor Immune Estimation Resource (TIMER 1.0, https://cistrome.shinyapps.io/timer/) (18).

### Distribution of Immune-Related Hub Genes in TME of GBM From Single-Cell Data

Data for the single cell GBM analysis was derived from the paper “An Integrative Model of Cellular States, Plasticity, and Genetics for Glioblastoma”, and the Seurat R package was used to reprocess the count matrix in which the dimensional reduction plot and cell type annotation were both retrieved from published meta data (19). The distribution of expressions of the hub genes was created using the Feature Plot function.

## Results

### Data Source and Identification of Survival-Related Immune Cells

**Figure 2:**
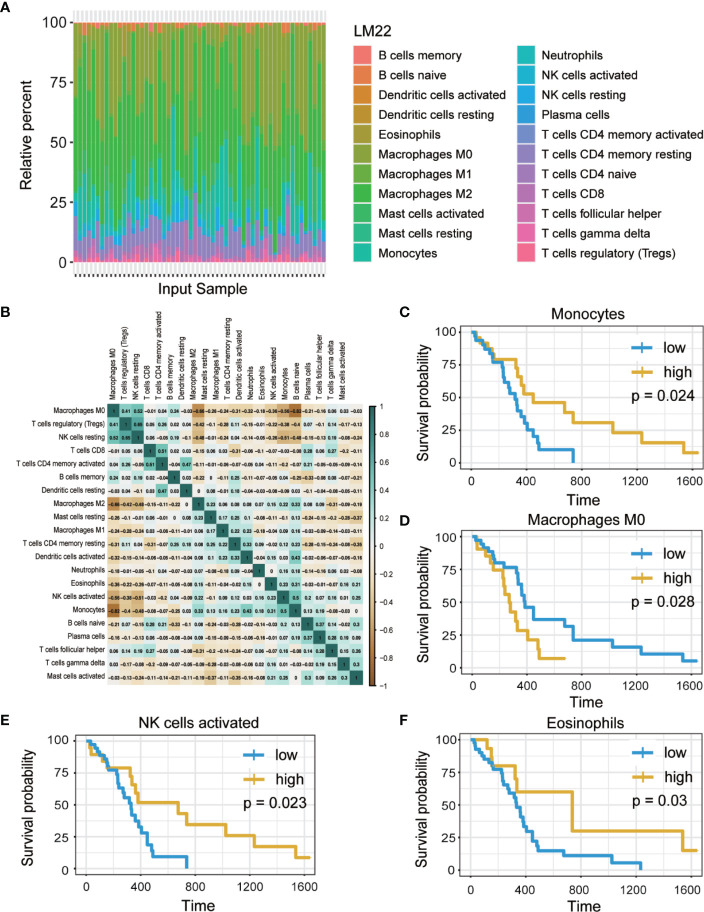
The abundance ratios of 22 immune cells and overall survival analysis. **(A)** The abundance ratios of immune cells in the 57 samples. The specific 22 immune cells corresponded to to one sample by different colors as shown in barplot. **(B)** The abundance ratios matrix of 22 immune cells. The value represents the correlation value, green represents the positive correlation while brown represents negative correlation. **(C**–**F)** Overall survival analysis of four immune cells based on Kaplan Meier-plotter from the comparison of groups of high (yellow line) and low (blue line) genes expression. (p<0.05).

The workflow of the study is presented in **Figure 1**. Publicly available data for the 166 cases of GBM, including RNA-Seq (FPKM and counts format) and clinical data were downloaded from TCGA database. The abundance ratios of 22 immune cells in the 57 samples are presented in the **Figure 2A**, and the relationship between abundance ratios of the immune cells is presented in **Figure 2B**. Consequently, the correlations between abundance ratios of immune cells were analyzed using Kaplan-Meier survival analysis to elucidate the potential role of the abundance ratios of immune cells in overall survival. The four immune cells that were associated with survival are presented in **Figures 2C–F**. The results of survival analyses indicated that there was a significant negative correlation between M0 Macrophages, while monocytes, activated NK cells, and eosinophils predicted positive overall survival.

### Clinical Data Correlated With Survival-Related Immune Cells

**Figure 3:**
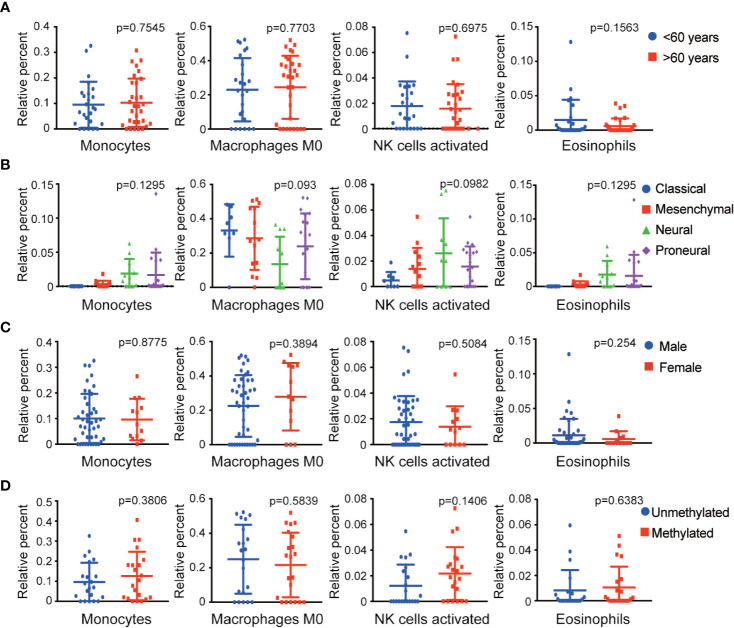
Relationship between four survival-related immune cells and clinical features. **(A–D)** The relationship between four survival-related immune cells and age, gender, expression subclass, and MGMT status.

To determine the effect of immune cells on the clinical characteristics of GBM, relevant GBM clinical data were downloaded to investigate correlation with the abundance ratios of survival-related immune cells. The clinical characteristics included age, gender, expression subclass, and MGMT promoter status. The odds ratio of monocytes and eosinophils increased in neural and proneural types and was higher in males than in females (**Figure 3**).

### Screening of Immune-Related Genes

**Figure 4:**
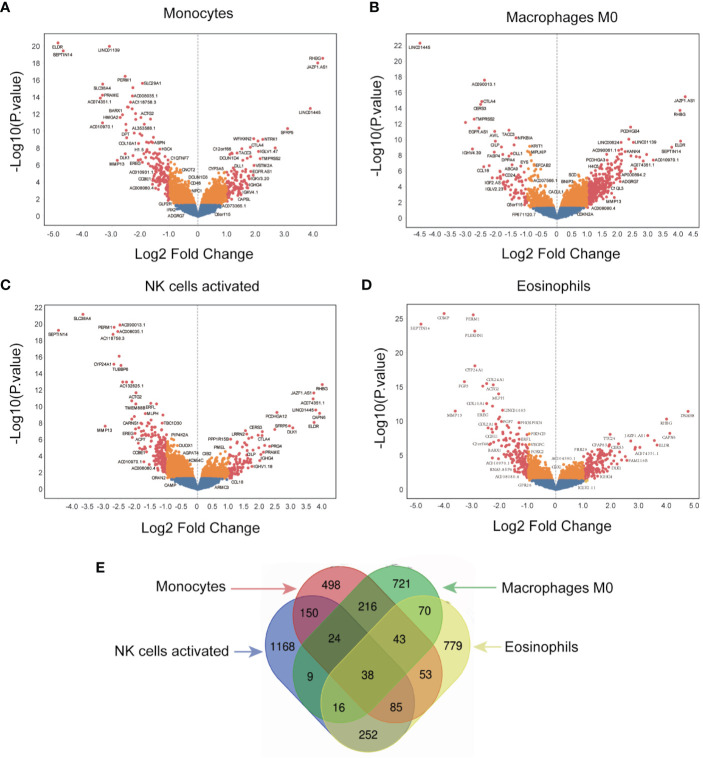
Screening for immune-related genes. **(A–D)** The volcano plot of all quantified genes in the analysis of monocytes, macrophages M0, NK cells activated, and eosinophils. **(E)** Venn diagram indicates the overlap of differentially expressed genes across the four different immune cells.

The immune-related genes were categorized into high- and low-expression groups in GBM to identify genes that associated with the four survival-related immune cells. Unique genes expression profiles of the four survival-related immune cells are presented by volcano plots in **Figure 4**. A total of 1,107 genes were identified in monocytes, 1,137 genes in macrophages M0, 1,742 genes in activated NK cells, and 1,336 genes in eosinophils (**Figures 4A–D**). In addition, 38 identical genes expressed in infiltration of the four immune cells are presented by Venn diagrams in **Figure 4E**.

### Functional Enrichment Analysis of Immune-Related Genes

**Figure 5:**
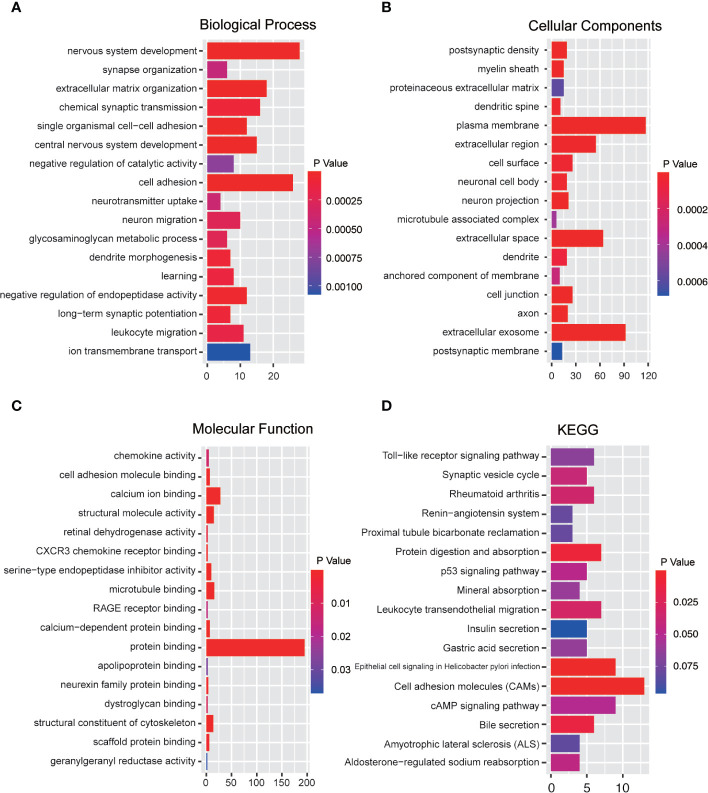
Functional enrichment analysis of immune-related genes. **(A)** Biological process analysis. **(B)** Cellular components analysis. **(C)** Molecular function. **(D)** KEGG pathway analysis (p < 0.05).

Functional enrichment analysis of immune-related genes was performed *via* DAVID website to reveal the potential functions of immune-related genes (**Figure 5**). GO term analysis revealed that immune-related genes were significantly enriched in the biological processes (BP) of nervous system development, cell adhesion, extracellular matrix organization, and chemical synaptic transmission (**Figure 5A**). Genes in the cellular components (CC) groups (**Figure 5B**) were primarily enriched in the plasma membrane, extracellular exosome, extracellular space, and extracellular region; the molecular functions (MF) were enriched in protein binding, calcium binding, structural constituent of cytoskeleton, and microtubule binding (**Figure 5C**). Moreover, the KEGG analysis revealed that immune-related genes were linked to cell adhesion molecules, cAMP signaling pathway, leukocyte transendothelial migration, protein digestion and absorption, and Toll-like receptor signaling pathway (**Figure 5D**). These results demonstrated that the genes were associated with the extracellular matrix of tumor microenvironment and cellular interaction.

### Modular Analysis Based on PPI Network

**Figure 6:**
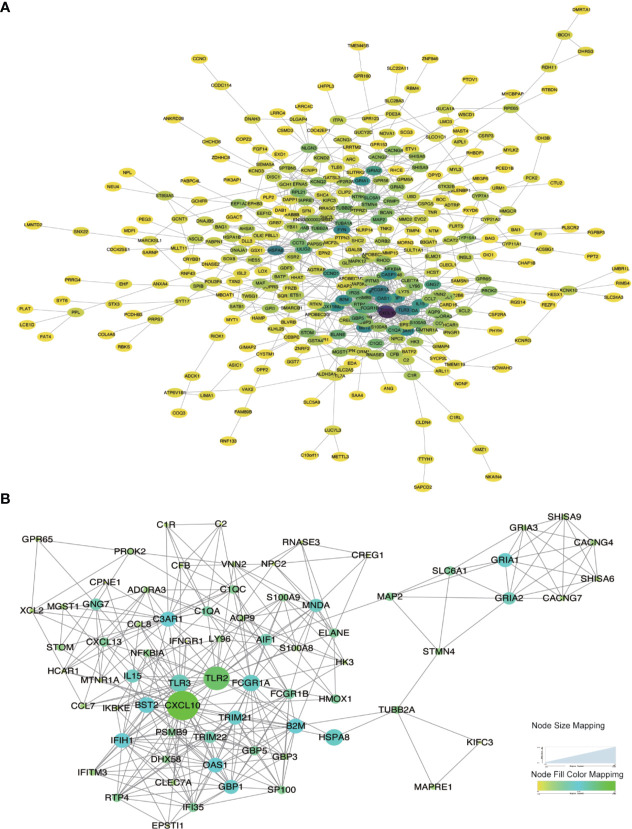
Protein-Protein interaction network construction and modular analysis. **(A)** PPI network was constructed using a total of DEGs. **(B)** The most significant module was marked. The color of a node reflects the log(Fc) value of the gene expression, the size of a node suggests the numbers of interacting genes with others.

Considering the limitation of the PPI networks regarding the number of genes, we screened all the differentially expressed genes but selected the genes only co-expressed in at least two immune cells. Overall, we identified 920 genes from 4,122 genes. These genes were imported into the online STRING tool to elucidate the interaction of immune-related genes. Finally, we got the PPI network with 357 genes which the combined-score was set to ≥ 0.4 (**Figure 6A**). We selected the most significant module for further functional enrichment analysis (**Figure 6B**).

### Identification of Survival-Related Hub Genes

**Figure 7:**
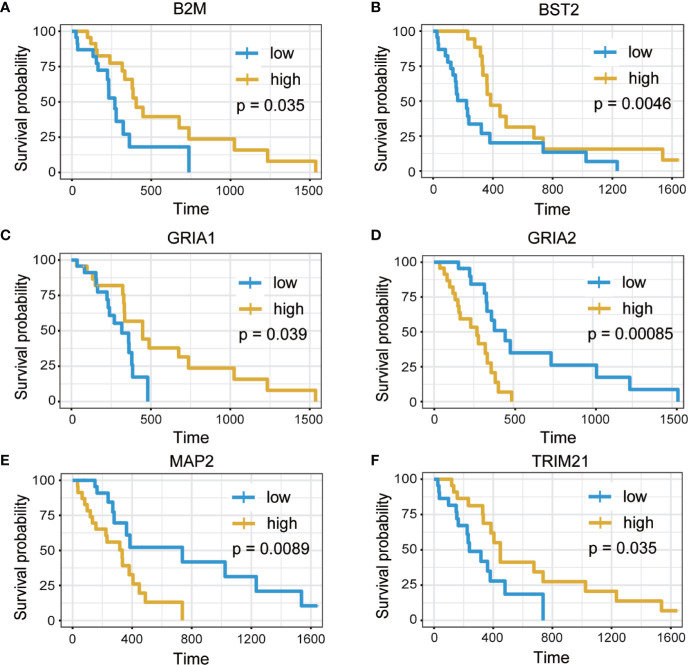
Overall survival analysis of six hub genes. **(A)** B2M. **(B)** BST2. **(C)** GRIA1. **(D)** GRIA2. **(E)** MAP2. **(F)** TRIM21 (p < 0.05).

**Figure 8:**
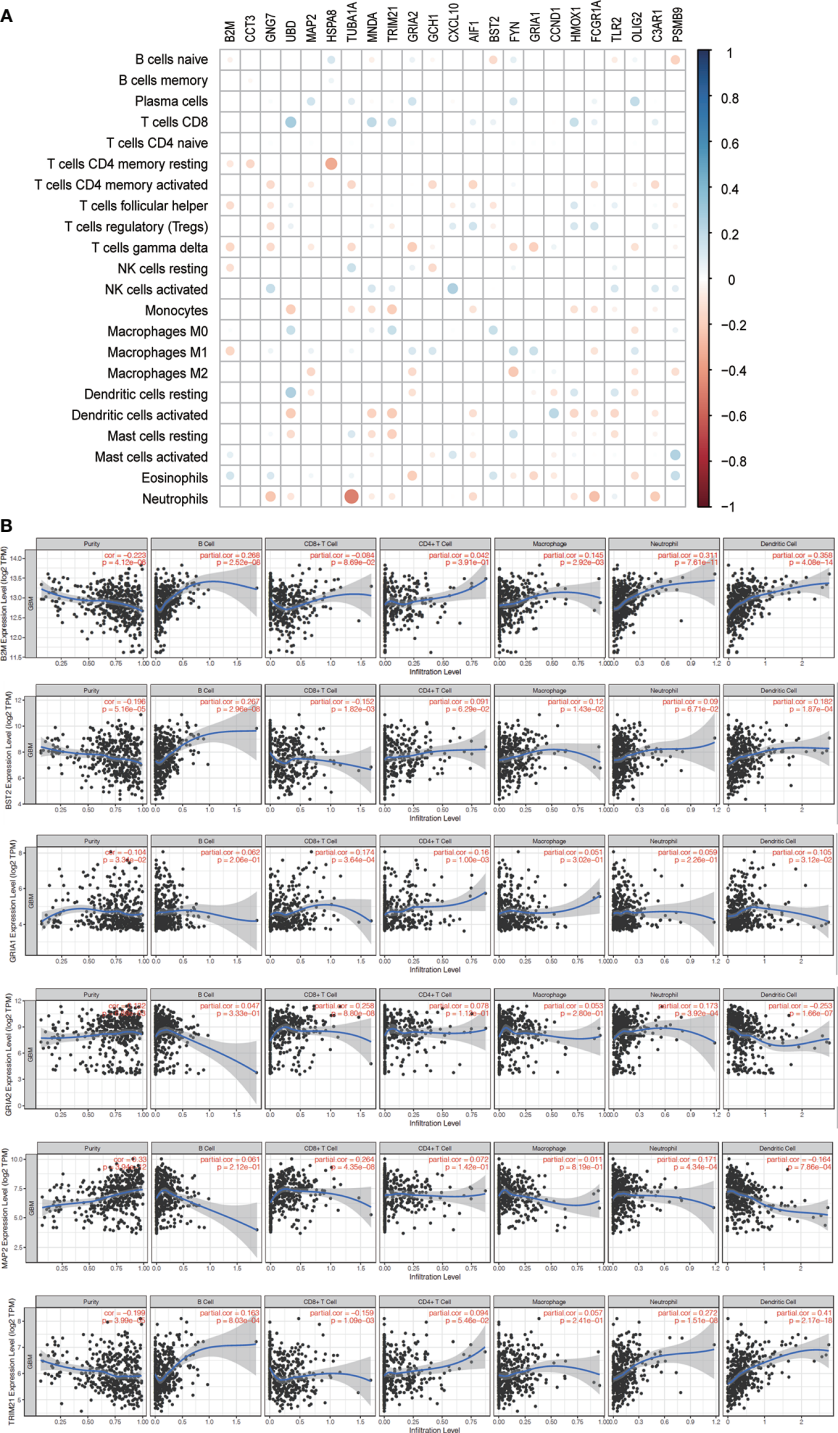
Immune infiltration of survival-related genes. **(A)** The correlation between expression proportion of hub genes and immune cells. Red suggests the positive correlation while the blue represents negative correlation. The size of point indicates P-value, and the color reflects the correlation. **(B)** The correlation analysis between survival-related genes and tumor infiltrating immune cells was performed. Scatter plots were generated with partial Spearman’s correlation and statistical significance.

A total of 24 hub genes with high connectivity in the modules were identified from the PPI network based on the cut-off criteria. We subsequently elevated the biological enrichment analysis of the 24 hub genes using the online tool (http://www.ncbi.nlm.nih.gov/gene) (**Table 1**). Six of the hub genes were significantly correlated with survival (**Figure 7**). GRIA1, BST2, B2M, and TRIM21 were positively correlated with the overall survival. GRIA2 and MAP2 were correlated with poor prognosis. The relationship between 24 hub genes and 22 immune cells analyzed using Person’s correlation analysis is performed in **Figure 8A**. The remarkable relationship between infiltration levels of immune cell types and survival-related hub genes was validated in TIMER. The results indicated that infiltration levels of CD8+ T cells, neutrophils, and dendritic cells were significantly associated with GRIA1, GRIA2, and MAP2 (**Figure 8B**). Furthermore, BST2 and B2M were correlated with B cells, macrophages, and dendritic cells, and TRIM21 was associated with B cells and neutrophils.

**Table 1:** The function of hub genes.

Number	Name	Full name	Function
1	FYN	FYN proto-oncogene, Src family tyrosine kinase	G-protein signaling_RhoA regulation pathway and Lipoprotein metabolism
2	HSPA8	Heat shock protein family A (Hsp70) member 8	ubiquitin protein ligase binding
3	CCND1	Cyclin D1	protein kinase activity and enzyme binding
4	GRIA1	Glutamate ionotropic receptor AMPA type subunit 1	PDZ domain binding and extracellularly glutamate-gated ion channel activity
5	TLR2	Toll like receptor 2	protein heterodimerization activity and transmembrane signaling receptor activity
6	B2M	Beta-2-microglobulin	identical protein binding
7	AIF1	Allograft inflammatory factor 1	calcium ion binding and actin filament binding
8	MAP2	Microtubule associated protein 2	structural molecule activity and calmodulin binding
9	OLIG2	Oligodendrocyte transcription factor 2	homodimerization activity and transcription factor activity, RNA polymerase II distal enhancer sequence-specific binding.
10	CXCL10	C-X-C motif chemokine ligand 10	signaling receptor binding and chemokine activity
11	GCH1	GTP cyclohydrolase 1	calcium ion binding and GTP binding
12	FCGR1A	Fc fragment of IgG receptor Ia	obsolete signal transducer activity, downstream of receptor and IgG binding
13	C3AR1	Complement C3a receptor 1	G protein-coupled receptor activity and complement component C3a receptor activity
14	TUBA1A	Tubulin alpha 1a	structural molecule activity
15	CCT3	Chaperonin containing TCP1 subunit 3	unfolded protein binding
16	HMOX1	Heme oxygenase 1	protein homodimerization activity and oxidoreductase activity
17	GNG7	G protein subunit gamma 7	obsolete signal transducer activity
18	C1R	Complement C1r	calcium ion binding and serine-type peptidase activity
19	BST2	Bone marrow stromal cell antigen 2	obsolete signal transducer activity
20	CYP19A1	Cytochrome P450 family 19 subfamily A member 1	iron ion binding and electron transfer activity
21	GRIA2	Glutamate ionotropic receptor AMPA type subunit 2	ionotropic glutamate receptor activity and AMPA glutamate receptor activity
22	MNDA	Myeloid cell nuclear differentiation antigen	Innate Immune System and Apoptosis and Autophagy
23	MAF	MAF bZIP transcription factor	DNA-binding transcription factor activity and DNA-binding transcription activator activity, RNA polymerase II-specific
24	TRIM21	Tripartite motif containing 21	identical protein binding and ligase activity

### Validation of the Correlation Between Immune Cell Infiltration and Survival-Related Hub Genes

**Figure 9:**
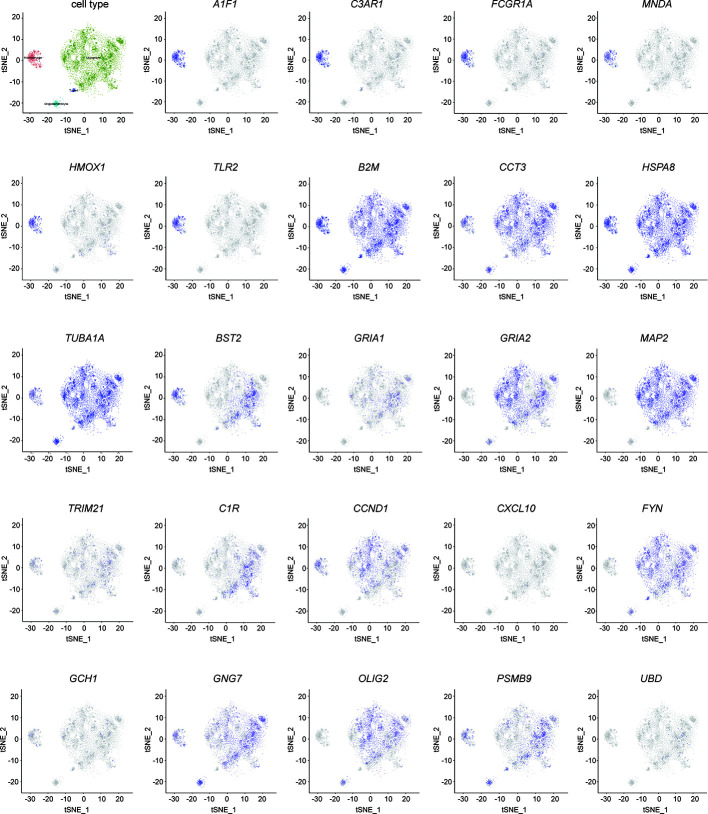
Validation of hub genes in single- cell sequencing in GBM. t-distributed neighbor embedding(tSNE) plot of all single cells. The color represents the expression of markers for Malignant cells (green), marcophages(magenta), oligodendrocytes (cyan), and T-cells (blue).

The correlation between survival-related hub genes and immune cell infiltration in GBM was analyzed after determining the prognostic value of hub genes (**Figure 9**).

In addition, gene expression data of immune cells for 134 GBM samples were downloaded from CGGA database to investigate the significance of immune cells identified from TCGA database. The results we obtained from CCGA revealed that activated NK cells (**Figure S1**, p = 0.019) and monocytes (**Figure S1**, p = 0.023) were associated with positive prognosis, which are consistent with the data we have gotten previously from the TCGA database (**Figure S1**).

### Validation of the Expression of Immune-Related Hub Genes by Single-Cell Sequencing

The cells were classified as malignant and non-malignant cell types by combining three approaches; high expression of markers classified as non-malignant cells such as macrophages, T cells, and oligodendrocytes. The distribution of hub genes expressions in the four cell clusters is displayed in **Figure 9**. *A1F1*, *C3AR1*, *FCGR1A*, *MNDA*, *HMOX1*, and *TLR2* were only expressed in macrophages. *B2M*, *CCT3*, *HSPA8*, and *TUBA1A* were significantly expressed in all the four cell clusters. With reference to survival-related genes, *BST2* was detected in macrophages, T-cells, and malignant cells. *GRIA1* and *GRIA2* were expressed in oligodendrocytes and malignant cells. *MAP2* was only detected in malignant cells. However, *TRIM21* was not detected in any of the cells types. Notably, microglia are the vital macrophages of the brain, and they act as the primary form of immune defense in the central nervous system. A specific microglial marker in humans, TMEM 119, was used to distinguish microglia from macrophages in the brain (**Figure S2**). We subsequently identified the expression of hub genes in microglia and found *AIF1*, *B2M*, *BST2*, *C3AR1*, *CCND1*, *CCT3*, *FCGR1A*, *GNG7*, *HMOX1*, *HSPA8*, *MNDA*, *TLR2*, and *TUBA1A* were significantly expressed (**Figure S2**).

## Discussion

The present study analyzed immune cells and immune-related genes in TME of GBM to establish a potential strategy for GBM immunotherapy. The study identified immune-related genes in TME, which significantly contributed to the survival of patients with GBM from TCGA database. Four survival-related immune cells were initially identified from GBM samples and the genes correlating to the levels of four immune cells analyzed. Furthermore, GO and KEGG enrichment analysis were conducted to investigate the biological functions of immune-related genes. Subsequently, all the immune-related genes were imported to construct a PPI network, and 24 hub genes obtained. Finally, the immune cell types in patients with GBM were validated using CGGA database, and hub genes validated in single-cell sequencing.

Four types of survival-related immune cells associated with GBM were identified from TCGA database, including M0 macrophage, monocytes, NK cells and eosinophils. Previous research has indicated that immune cells, especially tumor-associated macrophages (TAMs) in TME interact with tumor cells through direct contact or different signaling pathways. TAMs are crucial components of infiltrating immune cells, accounting for 30–40% of the cellular components in GBM (20). Immune cell populations in GBM are classified into two categories: microglia and bone marrow-derived monocytes. The BBB is damaged during tumor progression (21). With the accumulation of a family of monocyte chemoattractant family of proteins (MCPs), monocytes from the periphery infiltrate into the tumor across the BBB, and then differentiate into macrophages. Tumor-associated macrophages are often regarded as the facilitators of tumor proliferation due to their proangiogenic and immunosuppressive effects (21). M0 macrophages, which are referred to as ‘alternatively activated macrophages,’ can be polarized into M1 or M2 phenotypes by environmental signals (22). M1 macrophages can produce pro-inflammatory cytokines that are essential for host defense and exert tumoricidal effects in GBM (21). However, M2 macrophage phenotype is considerably involved in tumor cell proliferation and prediction of poor clinical prognosis in patients with GBM patients (23). M1 and M2 macrophages are plastic and heterogeneous immune cells, and the TME facilitates the regulation of functional polarization of TAMs (24). Currently, researchers have been working on promoting the reversal of TAMs from M2 to M1 based on their polarization (25, 26). Therefore, the results may indicate that the macrophages in TME of GBM could be used as potential therapeutic targets for GBM immunotherapy.

NK cells accounts for 2.11% of the total cellular components in GBM, which constitutes the lowest proportion of all immune cells infiltrating in GBM (27). NK cells have been reported to recognize target cells that are deficient in the surface expression of major histocompatibility complex (MHC) molecules, and can directly lyse tumor cells without prior activation (28). However, TME influences the immune function of NK cells and causes immune evasion. The upregulation of growth factor signaling pathways or the loss of cell cycle regulators promotes evasion of GBM from surveillance through resistance to NK-derived cytotoxicity (29). Moreover, GBM cells express high levels of MHC class I molecules and human leukocyte antigens (HLA)-A, HLA-B, and HLA-C ligands, which inhibit functions of NK cells *via* killer immunoglobulin-like receptors (KIRs) (30). Therefore, blocking KIRs could disrupt the tumor microenvironment and attenuate the activity of NK cells to kill GBM cells. Increasing the number of NK cells infiltrating the GMB microenvironment and modification of NK cells could be a potential treatment intervention for GBM (31, 32). Emerging evidence has demonstrated that the activation of eosinophils induces initiation, promotion and progression of GBM (33).

Previous advances have indicated the eosinophil-derived neurotoxin (EDN) and eosinophil cationic protein (ECP) play a critical role in preventing GBM initiation (34). During GBM promotion, eosinophils are activated by GBM mediators, which in turn lead to the production of tumor promoting growth factors (35). Nevertheless, the mechanisms of immune response in GBM remain indeterminate; therefore, further studies are required to investigate the mechanism involved.

More importantly, KEGG enrichment analysis indicated that these differential immune-related genes were enriched in the classical pathway, such as cell adhesion molecules (CAMs) and cAMP signaling pathway. CAMs are glycol-proteins expressed on the cell surface and play a critical role in multiple biologic processes during tumor development (36). It has been reported that CAMs mediate the process of immune responses in the tumor microenvironment, such as immune cell recruitment, immune cell activation, and formation of immunological synapse between immune cells and tumor cells (37). The cAMP signaling pathway, which acts as universal second messengers regulates pivotal physiological processes. The increases of intracellular cAMP inhibits innate immune functions (38). At the BP level, these differential immune-related genes were significantly enriched in cell adhesion, and extracellular matrix organization. In the CC groups, the differential immune-related genes were related to extracellular exosome, extracellular space, and extracellular region; the MF groups were enriched in protein binding, the structural constituent of cytoskeleton, and microtubule binding. Theses pathways are all related to the extracellular matrix components and cell’s cytoskeleton in the microenvironment. These above results further indicate the reliability of the immune differential genes and their relevance to the GBM tumor microenvironment.

Furthermore, we identified 24 hub genes, and 6 of these genes (GRIA2, GRIA1, BST2, MAP2, B2M, and TRIM21) have significant correlation with prognosis and were considered as predictive biomarkers that could provide valuable insights into new immunotherapy strategies. Previous studies have demonstrated that glioma cells can secrete excitotoxicity glutamate that mediates neuronal death in glioma microenvironment. Moreover, glutamate secretion promotes tumor expansion by inducing inflammatory response within the surrounding areas (39). Researchers have established that the expression of α-amino-3-hydroxy-5-methyl-4-isoxazolepropionic acid receptors (AMPARs) protects GBM cells from the glutamate-rich tumor microenvironment (40). AMPARs are complexes consisting of four subunits (GluR1, GluR2, GluR3, and GluR4). GRIA1 and GRIA2 are also referred to as GluR1and GluR2, respectively. Glutamate receptors (GluRs) are receptors that bind to glutamate, and they function as ligand-gated ion channels in the central nervous system and mediate transmission in excitatory synapses (41). The subunit composition of AMPARs depends on the conductance properties of Ca^2+^. Absence of GluR2 subunit promotes permeability to Ca^2+^, whereas presence of GluR2 inhibits permeability to Ca^2+^ (42). However, GluR1 and GluR4 subunits also function as Ca^2+^- permeable AMPARs. Ishiuchi et, al found GluR1 proteins were substantially expressed in most tumor cells, whereas GluR2 was mainly expressed in normal tissues in human glioblastoma samples (43). Furthermore, it has been suggested that blockage of Ca^2+^ influx through GluR2 expression suppresses migration and induces apoptosis in human glioblastoma cells (44). In addition, knocking down GluR1 inhibits glioma growth (45). Therefore, the conversion of Ca^2+^- permeable AMPARs to Ca^2+^- impermeable could be a potential therapeutic target for brain tumors (43). TRIM21 expression is correlated with prognosis, which acts as a tumor suppressor in patients with GBM (46). TRIM21 depletion in GBM enhanced cell proliferation and tumor growth. Lee et, al found that phosphofructokinase 1 (PFK1) expression promotes human glioblastoma progression, while TRIM21 exert anti-tumor effect by mediating poly ubiquitination and degradation of PFK1 (46). Therefore, TRIM21 is a novel target for glioblastoma treatment.

The expression of 24 hub genes in human glioblastomas was validated using single-cell sequencing. Conventional RNA-seq is regularly performed on a bulk level and only measures the average gene expression based on mixed cell populations in samples. Genes that contribute to cell-by-cell variations cannot be detected using conventional RNA-seq data of GBM downloaded from TCGA database (47). However, single-cell RNA-seq (scRNA-seq) profiles for intracellular transcriptome at individual cell level can reveal potential heterogeneous tumors and the composition of glioblastoma tumor microenvironment (48). ScRNA-seq can easily identify highly variable genes in all cell types in the TME of GBM, including the two primary cell types: microglia/macrophages and oligodendrocytes, which are limited in conventional RNA-seq (49). For example, as we have mentioned above, the results of ScRNA-seq revealed that GluR1 and GluR2 were expressed in oligodendrocytes and malignant cells. The expression of Ca2+-permeable GluR confers protection against excitotoxicity and promotes progression of tumor (50). BST2 expression increases in the malignant cells of glioma during tumor progression (51). TLR2 expressed in microglia can promote glioblastoma progression by up-regulating the expression of MT1-MMP in microglia (52). The expression of CCND1 in microglia cells contributes to the differential diagnosis of oligodendrogliomas (53). The use of scRNA-seq to detect the expression of hub genes could significantly help us to accurately understand the function of hub genes in each cell (54). In addition, scRNA-seq demonstrates transcriptional heterogeneity associated with spatial specificity in distinct TME patterns (55). ScRNA-seq has emerged as a revolutionary tool to enhance our understanding of the profiles of hub genes in GBM, and offers insights with implications for both targeted and immune therapies for GBM (49).

In summary, the study identified four types of survival-related immune cells from TCGA database and 24 TME-related hub genes in glioblastoma. The correlation between immune cells and hub genes in patients with GBM was validated using single-cell sequencing data. The results revealed that the hub genes are involved in the development and progression of GBM. Therefore, the candidate genes identified in the study can be used as potential prognostic biomarkers for GBM. However, further studies on the immune cells and hub genes in GBM tumor microenvironment should be conducted to investigate the underlying mechanisms. The present study provides novel insights into the potential association between immune cell TME and GBM prognosis.

## Data Availability Statement

The datasets presented in this study can be found in online repositories. The names of the repository/repositories and accession number(s) can be found in the article/**Supplementary Material**.

## Author Contributions

SH, ZS, JS, and JP designed the study. SH, ZS, XH, TZ, and KH collected and analyzed data. SH, ZS, XH, QZ, and JP wrote the manuscript. All the authors approved the manuscript. All authors contributed to the article and approved the submitted version.

## Funding

This work was supported by the Natural Science Foundation of Zhejiang Province (project number: LZ20H090002) and the National Natural Science Foundational (project number: 82071285).

## Conflict of Interest

The authors declare that the research was conducted in the absence of any commercial or financial relationships that could be construed as a potential conflict of interest.

## Supplementary Material

Supplementary Figure 1 Overall survival analysis of 22 immune cells based on Kaplan Meier-plotter from the data of CGGA.

Supplementary Figure 2 Hub genes expression in microglia. Microglia classified from macrophages are marked in dark red, while hub genes are in orange.
