# Supplementary figures and images for: Identification of Immune Cell Infiltration and Immune-Related Genes in the Tumor Microenvironment of Glioblastomas
Source: Front Immunol. 2020 Oct 20;11:585034. doi: 10.3389/fimmu.2020.585034 (PMC7606992; doi:10.3389/fimmu.2020.585034)

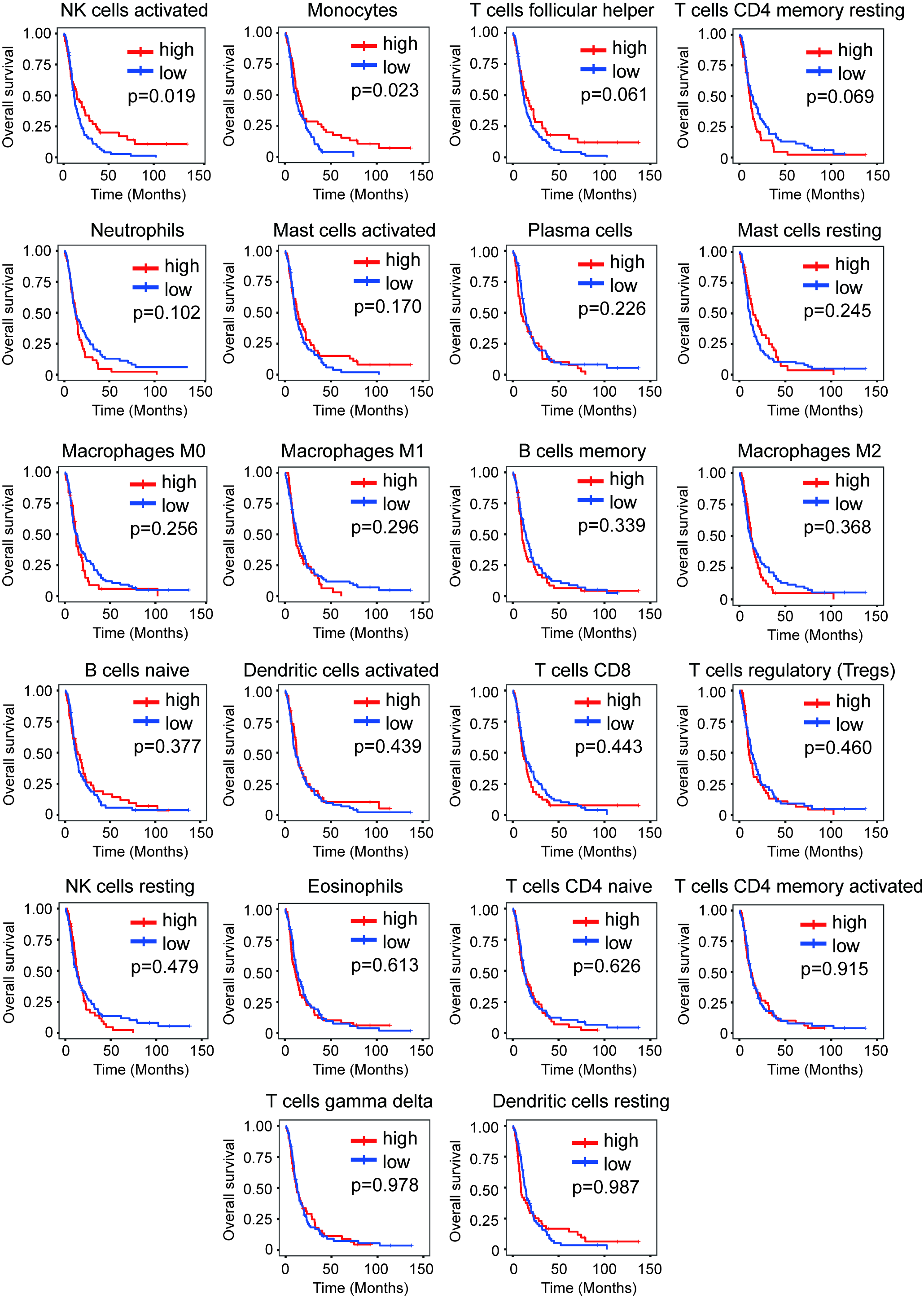

Supplement: Supplementary Figure 1 — Overall survival analysis of 22 immune cells based on Kaplan Meier-plotter from the data of CGGA. [file Image_1.tif]

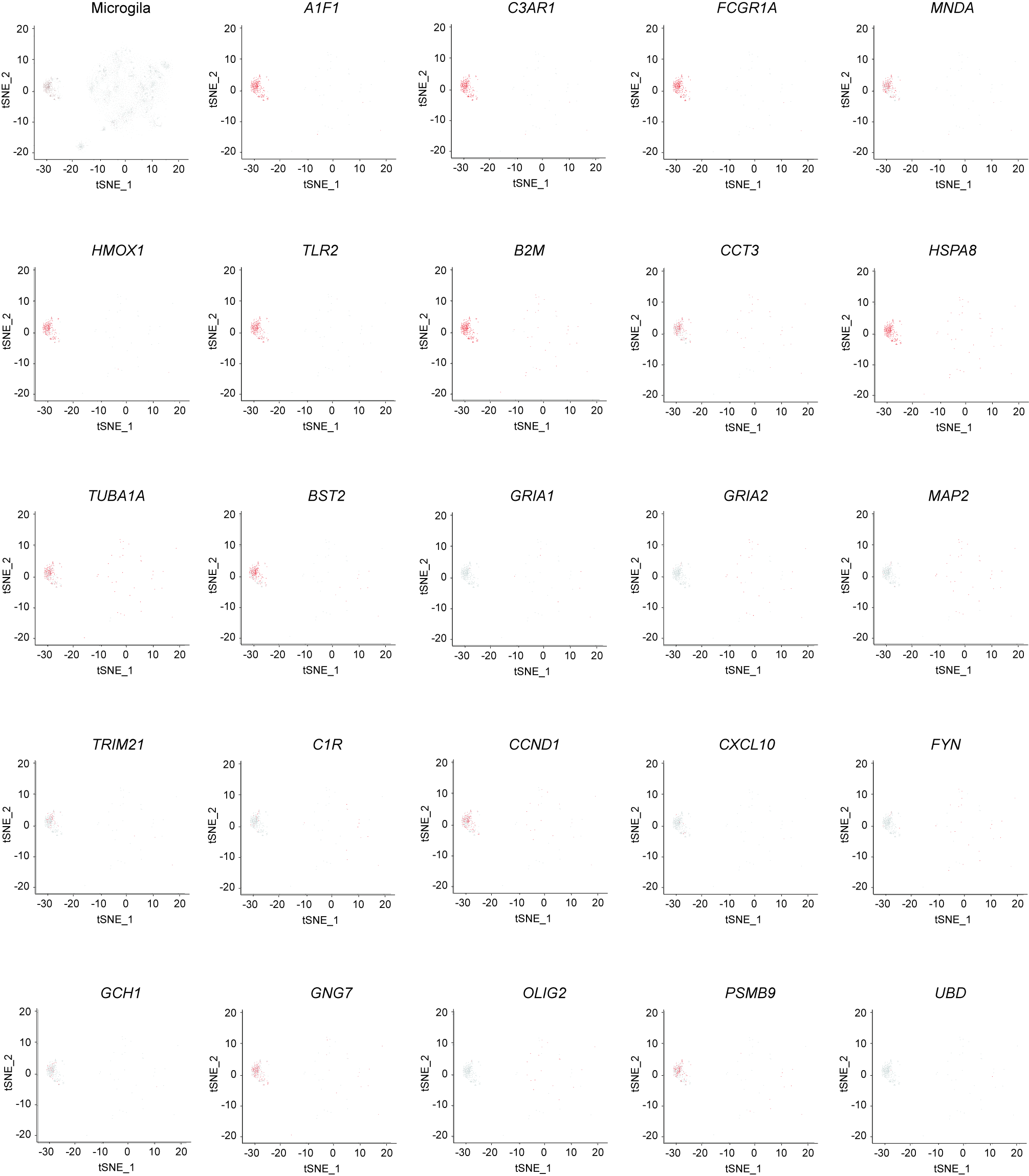

Supplement: Supplementary Figure 2 — Hub genes expression in microglia. Microglia classified from macrophages are marked in dark red, while hub genes are in orange. [file Image_2.tif]
